# Supplementary material for: Sensory coding and the causal impact of mouse cortex in a visual decision
Source: eLife. 2021 Jul 30;10:e63163. doi: 10.7554/eLife.63163 (PMC8324299; doi:10.7554/eLife.63163)
Supplement: Supplementary file 1. — Table lists the mice used in this study, sex/genotype information, and the number of sessions used for the data in each figure panel. [file elife-63163-supp1.docx]

|  | | | **Session count for each Figure** | | | | |
| --- | --- | --- | --- | --- | --- | --- | --- |
| **Name** | **Sex** | **Genotype** | **Fig. 2a-h** | **Fig. 2i-p *** | **Fig. 3a,d** | **Fig. 3g,h** | **Fig. 1e-h Fig. 4c,d** |
| Beadle | M | Ai32 x PV-Cre |  |  |  | 4 | 5 |
| Bovet | M | Ai32 x PV-Cre |  |  |  | 5 | 5 |
| Burnet | M | Ai32 x PV-Cre |  |  |  | 14 | 4 |
| Kornberg | M | Ai32 x PV-Cre |  |  |  | 14 | 8 |
| Medawar | M | Ai32 x PV-Cre |  |  |  | 14 | 4 |
| Ochoa | M | Ai32 x PV-Cre |  |  |  | 14 | 8 |
| Chomsky | F | Ai32 x PV-Cre |  |  | 16 |  |  |
| Morgan | F | Ai32 x PV-Cre |  |  | 8 |  |  |
| Murphy | M | Ai32 x PV-Cre |  |  | 28 |  |  |
| Spemann | M | Ai32 x PV-Cre |  |  | 26 |  |  |
| Whipple | F | Ai32 x PV-Cre |  |  | 13 |  |  |
| Kendall | F | Ai95 x VGlut1-Cre | 2 |  |  |  |  |
| Moniz | M | Ai95 x VGlut1-Cre | 9 | 3 |  |  |  |
| Muller | F | Ai95 x VGlut1-Cre | 1 | 3 |  |  |  |
| Theiler | F | Ai95 x VGlut1-Cre | 1 | 1 |  |  |  |
| Forssman | M | C57BL/6J |  | 4 |  |  |  |
| Richards | M | C57BL/6J |  | 5 |  |  |  |
| Chain | M | Snap25-GCaMP6s | 4 |  |  |  |  |
| Radnitz | F | Snap25-GCaMP6s | 6 | 5 |  |  |  |
| Cori | F | tetO-G6s x CaMK2a-tTA | 6 | 3 |  |  |  |
| Hench | M | tetO-G6s x CaMK2a-tTA | 7 | 4 |  |  |  |
| Reichstein | M | tetO-G6s x CaMK2a-tTA | 3 |  |  |  |  |
| Lederberg | F | Vglut1-IRES2-Cre-D |  | 7 |  |  |  |
| Tatum | F | Vglut1-IRES2-Cre-D |  | 4 |  |  |  |

**Supplementary Table 1: Mouse genotype and session count for each figure**

* Data previously published in Steinmetz, N.A., Zatka-Haas, P., Carandini, M., Harris, K.D., 2019. Distributed coding of choice, action and engagement across the mouse brain. Nature 1–8.
